# Causal evidence of the association between green and blue spaces (GBS) and maternal and neonatal health: a systematic review and meta-analysis protocol

**DOI:** 10.1136/bmjopen-2023-082413

**Published:** 2024-08-07

**Authors:** Rukun K.S. Khalaf, Selin Akaraci, Faye D. Baldwin, Rebecca S. Geary, Ruwanthi Kolamunnage-Dona, Ruth F. Hunter, Sarah E. Rodgers

**Affiliations:** 1 Institute of Population Health, University of Liverpool, Liverpool, UK; 2 Centre for Public Health, Queen's University Belfast, Belfast, UK

**Keywords:** PUBLIC HEALTH, Systematic Review, Maternal medicine, Pregnancy, NEONATOLOGY

## Abstract

**ABSTRACT:**

**Introduction:**

Previous systematic reviews investigating the effects of green and blue space (GBS) on maternal and neonatal health have mainly focused on cross-sectional evidence, limiting potential causal inferences. The last review on the topic was published in January 2024. This review focused on residential greenness effects and neonatal health only but did not include other green/blue space measures, or maternal health outcomes. This review also only included papers published up to June 2023; discounting the 15 studies that have been published since. Thus, this study will capture the growing number of studies that generate causal evidence and aims to investigate the association between GBS and maternal and/or neonatal health.

**Methods and analysis:**

The study protocol was developed with reference to the Preferred Reporting Items for Systematic Reviews and Meta-Analyses guidelines. This review will include study designs such as experiments, quasi-experiments, longitudinal studies and more. The study independent variable must be a GBS, green space and/or blue space measure. Eligible maternal health outcomes are those reported during pregnancy and up to 1 year after pregnancy. Neonatal health outcomes are limited to neonates no older than 28 days. A total of seven online databases will be searched: Medline, Scopus, Web of Science, PsycInfo, Embase, Environment Complete, and Maternity and Infant Care Database. Abstract and full-text screenings will be undertaken by three reviewers. Risk of bias assessment will be conducted based on the Risk of Bias in Non-randomized Studies-of Exposure framework.

A narrative synthesis will be undertaken. If sufficiently comparable studies are identified, meta-analyses using random effects models will be conducted. We will explore heterogeneity using the I^2^ test.

**Ethics and dissemination:**

Ethical approval is not required as all the data will be derived from published primary studies that have already obtained ethical permissions. The findings will be disseminated through relevant conferences and peer-reviewed publications.

**PROSPERO registration number:**

CRD42023396372.

**Strengths and limitations of this study**

- This systematic review will serve as a much-needed expansion on recent reviews to include the variety of green/blue space measures and maternal health studies.
- The focus on causally designed studies will allow for more confident causal inferences between green and blue space (GBS) exposure and maternal and/or neonatal health outcomes compared with alternative study designs.
- The ability to conduct a meta-analysis may be hindered by the variety of eligible maternal and neonatal health outcomes and GBS exposures.
- The dearth in blue space studies may limit our ability to develop a summary effect estimate of blue space exposure against a maternal and/or neonatal outcome.

## Introduction

 Our environment plays an important role in shaping and influencing our health. As a result, policy stakeholders have increasingly stressed the importance of considering environmental factors in public health decision-making. Research investigating how green and blue space (GBS) affects health conditions such as mental well-being has motivated this growing interest.^1^ Among children, living closer to a blue space has been associated with lower levels of obesity and improved psychosocial functioning.^2^ This is crucial as international and national policies have highlighted the importance a good start in the first 1000 days of life can have on one’s health across the lifespan.^3 4^

It is hypothesised that GBS affects health via three pathways.^5^ These include instoration, restoration and mitigation. Instoration refers to physical activity and community cohesion. Restoration refers to mental restoration including stress relief. The mitigation pathway refers to GBS blunting effects on harmful environmental risks such as air pollution. In this way, GBS exposure can positively affect maternal and neonatal health.

Indeed, in 2020, two systematic reviews illustrating the associations between GBS and birth outcomes and the effects of green space exposure on adverse maternal outcomes were published.^6 7^ Both reviews found a positive association between green space and birth weight. Neither study found evidence of an association between green space and preterm birth. Additionally, the review that included blue space exposures did not yield conclusive evidence of an association with pregnancy outcomes.^6^ Since then, no recent systematic reviews on the impact of blue space exposure on neonatal and/or maternal health have been conducted.

The latest meta-analysis found increased birth weight following maternal exposure to greenness.^8^ Since then, many studies using causal methods have been published. Such studies include Chipman *et al* that carried out a prospective study to investigate the impact of greenness on birth outcomes in rural USA.^9^ Sun *et al* explored perinatal depression and green space exposure in a retrospective cohort study.^10^ Thus, an updated review examining GBS measures and their impact on both maternal and neonatal health outcomes is needed.

Recent studies have indicated protective effects of green space exposure on maternal outcomes such as pre-eclampsia and birth outcomes including preterm birth.^11 12^ However, the evidence is mixed. Studies have found close to null significant associations between green space exposure and term birth, size for gestational age and birth weight.^13 14^ Other studies have found that maternal green space exposure was not associated with gestational age, preterm birth or pre-eclampsia.^15 16^

Similar studies conducted in the USA indicated that, for every one IQR increase in maternal residential green space exposure within a 250 m buffer around a mother’s home, the HR for reported preterm birth was 0.978 (95% CI 0.973 to 0.983).^12^ Studies have also reported stronger associations between green space exposure (50 m buffer) and higher birth weight (β=38.9 (95% CI 13.6 to 64.3) for every one interquartile increase in average greenness, p=0.003) among mothers who reported lower socioeconomic status levels than those living in less deprived areas.^17^ There is also compelling evidence to suggest that the effects of GBS on health differ according to when exposure occurred among the gestational trimesters.^12^

With such varied results in the field, it is vital that a systematic review and meta-analysis is undertaken to clearly summarise and understand the scientific findings on the topic. Moreover, studies often employ divergent measures of GBS/green space/blue space that can affect reproducibility and generalisability of findings. Existing reviews also incorporate evidence from cross-sectional studies, which does not support causal inferences. Additionally, the last review was limited to papers published up to June 2023, at least 15 papers on the topic have been found since.^8^

Therefore, this systematic review aims to critically appraise existing studies that employ causal methodologies in studying the impacts of GBS on maternal and neonatal health. Using the Population, Exposure, Comparator, Outcome and Study design (PECOS) framework, this review intends to answer the question, ‘What is the causal evidence of an association between GBS and maternal and neonatal health outcomes?’.

This review forms part of an ongoing study (2022–2026) that will investigate the association between GBS on maternal and neonatal health in Wales, Merseyside and Cheshire.

## Methods and analysis

This reporting of this protocol was developed using the Preferred Reporting Items for Systematic Review and Meta-Analysis Protocols checklist (online supplemental file 1).^18 19^

### Inclusion and exclusion criteria

This review will focus solely on studies that employ causal methods to examine the association or effects of GBS on maternal and/or neonatal health. Included study designs are experiments, quasi-experiments, cohort studies, case–control studies, longitudinal studies and studies that conducted mediation analysis. Prospective cohort studies, experiments and quasi-experiments (eg, natural experiments) will provide strong causal evidence because exposure precedes the outcomes.^20^ Case–control and retrospective longitudinal studies will be included as the temporal sequence of exposure and outcomes can be established.^20^

Included studies will not be limited by country or time. Papers will be restricted to those published in English. Papers must have been peer reviewed and published in scientific journals.

The PECOS framework will be used to establish the overall study inclusion criteria (table 1). Green and blue space will be defined as either green space, blue space or GBS. At least one measure of GBS must be included as an independent variable. The interpretation and methods of measuring the exposure are subject to the individual study and will be evaluated for risk of bias. The studies must ensure that sufficient explanation is supplied on the definition and methods of GBS measures.

**Table 1:** Participant, Exposure, Comparator, Outcome and Study design (PECOS) criteria used in the systematic review

Alphabet	Corresponding term	Study term
P	Population	Pregnant women with singleton births. Neonates.
E	Exposure	Physical and/or visual exposure to green space, considering both its quantity and quality. Physical and/or visual exposure to blue space, considering both its quantity and quality. Physical and/or visual exposure to both GBS, considering both their quantity and quality.
C	Comparator	Those exposed to the lowest level of GBS. Those who are not exposed to GBS.
O	Outcome	Pregnancy complications. Pregnancy-related outcomes within the postpartum period (up to 1 year after gestation). Birth outcomes.
S	Study design	Experiments. Quasi-experiments. Cohort studies. Case–control studies. Longitudinal studies. Studies with mediation analysis.

GBSgreen and blue space

Grey literature (ie, book chapters) will be excluded. Studies that use a cross-sectional study design will be excluded. The study population must be limited to mothers or pregnant women who are legally classified as adults in the country of study, along with their respective newborns. Under-age mothers will be excluded from our review due to their higher risk of for pregnancy complications.^21^

### Exposures

Exposures will centre around any green space, blue space and/or GBS. Green spaces are areas that contain any vegetative greenery. Blue spaces are areas that contain a body of water regardless of size, type or depth. The studies will measure the physical and/or visual aspects of these spaces. For instance, included studies could measure the greenness of a space. The exposure variables must be measured before the occurrence of pregnancy outcomes (ie, during pregnancy rather than at time of birth). This review will include studies that measured exposures at multiple pregnancy time points (ie, trimesters). This review will exclude studies that employ virtual reality exposure techniques. Exposures that are measured using remote sensing imaging data (eg, Normalized Difference Vegetation Index (NDVI)) will be included because this often denotes surrounding exposure to GBS.^22^ Measures of GBS mobility will be based on accessibility metrics such as distance to a space and visits to a GBS.^23^

### Outcomes

Outcomes will include at least one maternal and/or neonatal health outcome. Neonatal health outcomes will be limited to neonates from the time of their birth to 28 days following birth.^24^ Maternal health outcomes will be limited to pregnancy complications during the peripartum period (the gestational period and immediately following birth) and the postpartum period (up to 1 year following birth). Studies whereby the main outcomes are non-pregnancy-related health conditions (eg, hypertension) reported among pregnant women or mothers will be excluded. Included studies must have comparator groups of mothers that are exposed to the comparatively lowest level of GBS measures.

### Search strategy and terms

Comprehensive electronic searches of Scopus, Web of Science, Environment Complete, PsycInfo, Embase, Medline, and Maternity and Infant Care Database will be conducted.

Keywords included in searches will be green blue space, green space, blue space, pregnancy complications, maternal outcomes, pregnancy outcomes and birth outcomes. These terms were chosen because target studies that we aimed for inclusion in this review have listed these keywords. The piloted test searches conducted with these terms yielded accurate and desired articles for this study. Search terms were derived from previous systematic reviews.^25^,^28^ Keywords were also recommended by coauthors (SR, RG, RH and RK-D) who are subject experts. The final search strategy can be found in online supplemental file 2.

Citation searching among included studies will be conducted. From this, references in target papers may yield additional articles missed in the initial screening.

#### Final search strategy

An example search strategy for the Scopus database is provided in table 2.

**Table 2:** Final search strategy for Scopus database

Limitations	Terms/criteria
Document type	Article
Language	English
Search terms	((TITLE-ABS-KEY (‘green blue space*’ OR ‘natural environment*’ OR ‘blue green space*’ OR ‘green and blue outdoor space*’ OR ‘blue and green outdoor space*’ OR ‘outdoor space*’)) OR (TITLE-ABS-KEY (‘blue space*’ OR blueness OR ‘water bod*’ OR waterfront* OR ‘blue area*’ OR ‘aquatic place*’ OR ‘coast*’ OR ‘beach*’ OR ‘lake*’ OR ‘wetland*’)) OR (TITLE-ABS-KEY (‘green space*’ OR greenness OR greenspace* OR ‘green infrastructure*’ OR greenery OR ‘green environment*’ OR ‘green land*’ OR ‘green roof*’ OR greenway* OR grassland* OR garden* OR ‘urban green’ OR ‘urban greenery’ OR vegetation OR ‘normalized difference vegetation index’ OR ‘enhanced vegetation index’ OR ‘leaf area index’ OR ‘green corridor*’ OR ‘nature*’))) AND (TITLE-ABS-KEY (longitudinal OR cohort OR ‘natural experiment*’ OR prospective OR retrospective OR ecological OR panel)) AND (TITLE-ABS-KEY (pregnan* OR maternal* OR birth* OR preterm* OR gestation* OR fetal OR fetus* OR antenatal OR newborn* OR infant* OR perinatal))

We used three potentially relevant articles to test and build our search strategy, which had been a priori identified by RK and SA. Experienced university librarians were consulted in developing the search strategy.

### Screening process

Following deduplication in EndNote, title and abstract screening will be conducted separately by three reviewers (RK, SA and FB) using Rayyan and a Microsoft Excel sheet. Reviewers will look to include studies that examine the topic of interest and/or contain the required keywords. The assessment criteria for study inclusion will centre on the study type, population, exposures and outcomes (table 3). Reviewers will be blinded to the other’s article inclusion decisions to reduce acquiescence response bias. If a reviewer is unable to decide on a study, the paper will be brought forward for full-text screening.

**Table 3:** Study screening eligibility criteria

Study characteristics	Study eligibility questions
Type of study	Did the paper use a causal study design?
Participants	Are neonates included?
	Are pregnant people included?
Type of exposure (*at least one answer must be affirmative*)	Is green space measured?
	Is blue space measured?
	Is green and blue space (GBS) measured?
Type of outcome (*at least one answer must be affirmative*)	Is a birth outcome measured?
	Is a pregnancy complication measured?

Full-text screening by RK, SA and FB will follow using EndNote. The assessment criteria used in full-text selection will be those which were used in the initial screening (table 3). Any disagreements will be settled with discussion and the guidance of coauthors (SR, RH, RK-D and RG).

### Data extraction

The data extraction form that will be used will be piloted using three target articles by RK and SA. The information selected for extraction has been chosen with reference to the Cochrane Collaboration guidance.^29^ The selected papers will be equally divided among three independent reviewers (RK, SA and FB) for data extraction on Excel. A total of nine domain variables will be collected from each study (table 4). The study information that will be extracted includes study background (eg, setting), study design (eg, aims), timeframe (eg, publication year), populations (eg, number of participants), exposures (eg, exposure definition), outcomes (eg, outcome definition), covariates, statistical methodologies (eg, models used) and effect measures (eg, summary results). Descriptive and analytical data will be extracted from the selected studies.

**Table 4:** Data items to be collected

Domains	Target variables
Background	Article title, journal, author(s), country/city, country income level, study setting (eg, rurality)
Study design	Aims, study design
Study timeline	Year of publication, study exposure time period, study outcome time period
Study population/participants	Brief study population description, number of participants
Exposure	Exposure(s) name, exposure(s) definition, exposure(s) buffer zone used, exposure(s) buffer type, exposure(s) data source
Outcome	Outcome(s) name, outcome(s) data source, outcome(s) definition
Covariates	Covariates included
Statistical methodology	Statistical methods used (eg, models)
Effect measures	Summary result, reported effect measures, CIs, p values

Outcomes are categorised into maternal or neonatal health outcomes. Example outcomes that will be included are pre-eclampsia, preterm birth and more. The measures of outcome should be valid and reliable; however, this will be further assessed for risk of bias later. Overall effect measures of the GBS exposure against maternal or neonatal health will be recorded. It is expected that most studies will report more than one relevant exposure and/or health outcome. If outcomes from differently adjusted statistical models are reported, values from the most fully adjusted model will be recorded. Values from study subgroup analysis (against variables such as urbanicity) or effect modifiers will be recorded.

The study authors will be contacted via email for further information, if needed.

### Risk of bias assessment

Risk of bias will be assessed independently by RK, SA and FB by applying the Standard Quality Assessment Criteria for Evaluating Primary Research Papers and Risk of Bias in Non-randomized Studies-of Exposure (ROBINS-E) guide.^30 31^ Risk of study bias will be assessed on the outcome and study level. Meta-bias(es) will also be assessed using the same risk of bias assessment tool. The studies will be appraised on the confounders used, sampling methods, measurement error or misclassification of variables, completeness of reporting, treatment of missing data, sensitivity analysis and the reported discussions and conclusions. Example questions include: was the measured exposure likely to be misclassified or measured with error or non-differentially treated? Important covariates that should be accounted for are measures of socioeconomic conditions (eg, mother’s employment), urbanicity of residence and pollution (eg, NO_2_ readings).^32^,^34^ The final risk of bias scores will be categorised as low risk of bias, some concerns of bias, high risk of bias and very high risk of bias.

Any disagreements in bias classification will be resolved through discussion and consultation with a fourth reviewer (SR, RH, RK-D or RG) if necessary.

### Data synthesis

First, a descriptive synthesis of the data will be undertaken and presented in the text. The extracted data will be presented in a table to highlight the study authors, characteristics, outcomes, exposures, statistical methodologies and risk of bias score. We will use proportional symbol maps to visualise the number of studies conducted across countries. Furthermore, we will develop a taxonomy containing the various exposure and outcome variables used to facilitate improved connections between the findings of the included studies. For example, a part of the taxonomy will include the differing variables (eg, proportion of surrounding household greenery) across studies that were collected to indicate green space exposure. This way, the findings will be presented in a structured order which will enable readers to compare the findings more effectively.

Forest plots showcasing the results from each study will be included. To address potential heterogeneity in GBS/green space/blue space measures and in maternal and neonatal health outcomes, the forest plots will be segregated based on whether a GBS/green space/blue space was measured as well as what outcome was measured. We anticipate creating at least four plots in this review. For example, a forest plot showcasing the ORs of green space measures against maternal outcomes will be created while another forest plot showing the ORs of blue space measures against maternal outcomes will be created. Similarly, two forest plots displaying the effect measures of neonatal outcomes will be created to separately indicate green space and blue space measures.

Information will include study author, year, effect measure of interest and corresponding CIs. A narrative analysis will be carried out to spotlight key themes in the selected studies.^35^ For each outcome discussed in the review, the strength of the body of evidence will be reported alongside the value.

Due to the diversity in exposure measures, conducting a meta-analysis may not be feasible. If, however, at least three studies report comparable exposures and outcomes and are rated low to moderate risk of bias, a meta-analysis will be conducted. Effect estimates from both observational and experimental studies will be treated similarly and combined in our meta-analysis. Linear dose–response analysis will be carried out if exposures are reported with similar buffer sizes and outcomes of interest. Pooled effect measures (eg, ratios or beta coefficients) and their corresponding CIs will be combined using a random effects model and presented in forest plots. It is expected that these summary effect measures will correspond to 0.1 unit change in the exposure measure (eg, NDVI) against the study outcome of interest or in comparison between the categorised exposure variables. Subgroup meta-analysis will be grouped and carried out based on similar exposure variables and buffer sizes. For example, studies that measure preterm birth against green space using NDVI with related residential buffer sizes (eg, 200–300 m) will be pooled together. Accessibility measures such as distance to blue space within a buffer range will be treated similarly.

The I^2^ statistics tests will also be conducted to indicate interstudy heterogeneity and consistency in the pooled results.^36 37^ An I^2^ statistic above 50% will indicate significant heterogeneity.^37^ We will also alternately exclude studies one at a time in our meta-analysis to observe any changes in our summary values. Funnel plots will be used to showcase publication bias among the selected studies. All analyses will be done in RStudio.

### Planned study start and end dates

The study start date will be June 2023. Data extraction of selected studies will be completed in December 2023. Searches were last conducted in June 2024. Search dates for each database can be found in online supplemental file 2. Data synthesis will be carried out from February to July 2024. The planned study end date is August 2024.

### Patient and public involvement

Neither the public nor the patients were involved in the creation of this protocol. The findings will be disseminated to lay audiences.

## Discussion

This systematic review aims to critically appraise and synthesise causal evidence from existing studies on the association between GBS and maternal and/or neonatal health outcomes to better understand the relationship between GBS and maternal and neonatal health. We hope to do this by carrying out a meta-analysis. This study will serve as a necessary expansion of the most recent reviews on the topic conducted in 2020, 2021, 2023 and 2024.^6^,^826 38^ We expect most studies to have been implemented in high-income countries and to focus on green space measures. This is in line with past reviews on the topic and our knowledge of existing literature. This study is different from the most recent reviews because we will include maternal health outcomes, blue space exposures and other green space measures beyond residential greenness.

### Strengths and limitations

This paper will include up to 15 studies that have since been published following the last review.^8^ Furthermore, searches will be carried out in health and environmental research databases to make certain relevant studies are captured from a wide range of scientific disciplines.

Some limitations to this study include the exclusion of data from non-causal studies (ie, reviews, ecological studies). Additionally, the expected dearth in blue space research on health may hinder our efforts in conducting a meta-analysis to fully summarise this relationship; hence, we may only conduct a narrative synthesis of blue space research.

## Ethics and dissemination

Ethical approval is not required as the studies will be publicly available.

The final review findings will be of interest to stakeholders in a range of sectors including health, and dissemination to environmental agencies (eg, Natural Resources Wales) is planned. The findings of the study will also be published in a scientific journal.

## supplementary material

10.1136/bmjopen-2023-082413 online supplemental file 1

10.1136/bmjopen-2023-082413 online supplemental file 2
