# Supplementary material for: Causal evidence of the association between green and blue spaces (GBS) and maternal and neonatal health: a systematic review and meta-analysis protocol
Source: BMJ Open. 2024 Aug 7;14(8):e082413. doi: 10.1136/bmjopen-2023-082413 (PMC11331951; doi:10.1136/bmjopen-2023-082413)
Supplement: online supplemental file 2 [file bmjopen-14-8-s002.pdf]

Supplementary file 2: Search strategy

**Database**	**Search terms**	**Date last searched**
Medline	See below	26 June 2024
Maternity & Infant Care Database	See below	28 June 2024
PsycInfo	See below	28 June 2024
Embase	See below	28 June 2024
Scopus	(TITLE-ABS-KEY("green blue space*" OR "natural environment*" OR "blue green space*" OR "green and blue outdoor space*" OR "blue and green outdoor space*" OR "outdoor space*") OR TITLE-ABS-KEY("blue space*" OR blueness OR "water bod*" OR waterfront* OR "blue area*" OR "aquatic place*" OR "coast*" OR "beach*" OR "lake*" OR "wetland*") OR TITLE-ABS-KEY("green space*" OR greenness OR greenspace* OR "green infrastructure*" OR greenery OR "green environment*" OR "green land*" OR "green roof*" OR greenway* OR grassland* OR garden* OR "urban green" OR "urban greenery" OR vegetation OR "normalized difference vegetation index" OR "enhanced vegetation index" OR "leaf area index" OR "green corridor*" OR "nature*") AND TITLE-ABS-KEY(longitudinal OR cohort OR "natural experiment*" OR prospective OR retrospective OR ecological OR panel) AND TITLE-ABS-KEY(pregnan* OR maternal* OR birth* OR preterm* OR gestation* OR fetal OR fetus* OR antenatal OR newborn* OR infant* OR perinatal)) AND (LIMIT-TO (DOCTYPE,"ar")) AND (LIMIT-TO (LANGUAGE,"English"))	26 June 2024
Web of Science	**((**"green space*" OR greenness OR greenspace* OR "green infrastructure*" OR greenery OR "green environment*" OR "green land*" OR "green roof*" OR greenway* OR grassland* OR garden* OR "urban green*" OR "urban greenery" OR vegetation OR "normalized difference vegetation index" OR "enhanced vegetation index" OR "leaf area index" OR "green corridor*" OR “nature*” **(Topic)) OR ((**"blue space*" OR blueness OR "water bod*" OR waterfront* OR "blue area*" OR "aquatic place*" OR “beach*” OR “coast*” OR “lake*” OR “wetland*” **(Topic)) OR (**"green blue space*" OR "natural environment*" OR "blue green space*" OR "green and blue outdoor space*" OR "blue and green outdoor space*" OR "outdoor	26 June 2024

	space*" **(Topic))) AND (**longitudinal OR cohort* OR "natural experiment*" OR prospective OR retrospective OR ecological OR panel **(Topic)) AND (** pregnan* OR maternal* OR birth* OR preterm* OR gestation* OR fetal* OR fetus* OR antenatal OR newborn* OR infant* OR perinatal **(Topic)) AND (LA==”ENGLISH”)**	
Environment Complete	(("green space#" OR greenness OR greenspace# OR "green infrastructure#" OR greenery OR "green environment#" OR "green land#" OR "green roof#" OR greenway# OR grassland# OR garden# OR "urban green" OR "urban greenery" OR vegetation OR "normalized difference vegetation index" OR "enhanced vegetation index" OR "leaf area index" OR "green corridor#" OR “nature#”) OR ("blue space#" OR blueness OR "water bod#" OR waterfront# OR "blue area#" OR "aquatic place#" OR “beach#” OR “coast#” OR “lake#” OR “wetland#”) OR ("green blue space#" OR "natural environment#" OR "blue green space#" OR "green and blue outdoor space#" OR "blue and green outdoor space#" OR "outdoor space#")) AND (longitudinal OR cohort# OR "natural experiment#" OR prospective OR retrospective OR ecological OR panel) AND (pregnan# OR maternal# OR birth# OR preterm# OR gestation# OR fetal# OR fetus# OR antenatal OR newborn# OR infant# OR perinatal) AND Limit=”English”	26 June 2024

Medline search strategy

Set	Search Statement
1	pregnan*.ab,ti.
2	maternal*.ab,ti.
3	birth*.ab,ti.
4	preterm*.ab,ti.
5	gestation*.ab,ti.
6	fetal.ab,ti.
7	fetus*.ab,ti.
8	antenatal.ab,ti.
9	newborn*.ab,ti.
10	infant*.ab,ti.
11	perinatal.ab,ti.
12	1 or 2 or 3 or 4 or 5 or 6 or 7 or 8 or 9 or 10 or 11
13	"green space*".ab,ti.
14	greenness.ab,ti.
15	greenspace*.ab,ti.
16	"green infrastructure*".ab,ti.

17	greenery.ab,ti.
18	"green environment*".ab,ti.
19	"green land*".ab,ti.
20	"green roof*".ab,ti.
21	greenway*.ab,ti.
22	grassland*.ab,ti.
23	garden*.ab,ti.
24	"urban green".ab,ti.
25	"urban greenery".ab,ti.
26	vegetation.ab,ti.
27	"normalized difference vegetation index".ab,ti.
28	"enhanced vegetation index".ab,ti.
29	"leaf area index".ab,ti.
30	"green corridor*".ab,ti.
31	13 or 14 or 15 or 16 or 17 or 18 or 19 or 20 or 21 or 22 or 23 or 24 or 25 or 26 or 27 or 28 or 29 or 30 or "nature*".ab,ti.
32	blue space*.ab,ti.
33	"green blue space*".ab,ti.
34	"natural environment*".ab,ti.
35	"blue green space*".ab,ti.
36	"green and blue outdoor space*".ab,ti.
37	"blue and green outdoor space*".ab,ti.
38	"outdoor space*".ab,ti.
39	32 or 33 or 34 or 35 or 36 or 37 or 38
40	longitudinal.ab,ti.
41	cohort*.ab,ti.
42	"natural experiment*".ab,ti.
43	prospective.ab,ti.
44	retrospective.ab,ti.
45	ecological.ab,ti.
46	panel.ab,ti.
47	40 or 41 or 42 or 43 or 44 or 45 or 46
48	"blue space*".ab,ti.
49	blueness.ab,ti.
50	"water bod*".ab,ti.
51	waterfront*.ab,ti.
52	"blue area*".ab,ti.
53	"aquatic place*".ab,ti.
54	48 or 49 or 50 or 51 or 52 or 53 or "beach*".ab,ti. or "coast*".ab,ti. or "lake*".ab,ti. or "wetland*".ab,ti.
55	31 or 39 or 54
56	12 and 47 and 55
57	Humans/
58	56 and 57
59	limit 58 to English language

Maternity and Infant Care Database search strategy

Set	Search statement
1	green*space.ti,ab.
2	(open adj space).ti,ab.
3	(public adj space).ti,ab.
4	(park not parkin*).ti,ab.
5	greenness.ti,ab.
6	green*way.ti,ab.
7	(green adj infrastructure).ti,ab.
8	vegetation.ti,ab.
9	NDVI.ti,ab.
10	blue*space.ti,ab.
11	(water adj body).ti,ab.
12	coast*.ti,ab.
13	(blue adj infrastructure).ti,ab.
14	pond*.ti,ab.
15	water*way.ti,ab.
16	lake.ti,ab.
17	sea.ti,ab.
18	ocean.ti,ab.
19	wetland.ti,ab.
20	(matern* adj health).ti,ab.
21	pregnan*.ti,ab.
22	new*born.ti,ab.
23	"gestational age".ti,ab.
24	birth*weight.ti,ab.
25	"preterm birth".ti,ab.
26	"birth outcome*".ti,ab.
27	(infan* adj health).ti,ab.
28	"head circumference".ti,ab.
29	"gestational diabetes".ti,ab.
30	"postpartum depression".ti,ab.
31	"preeclampsia".ti,ab.
32	(pregnan* adj outcome).ti,ab.
33	(green adj space).ti,ab.
34	(blue adj space).ti,ab.
35	1 or 2 or 3 or 4 or 5 or 6 or 7 or 8 or 9 or 10 or 11 or 12 or 13 or 14 or 15 or 16 or 17 or 18 or 19 or 33 or 34
36	20 or 21 or 22 or 23 or 24 or 25 or 26 or 27 or 28 or 29 or 30 or 31 or 32
37	35 and 36

PsycInfo search strategy

Set	Search strategy
1	green*space.ti,ab.
2	(open adj space).ti,ab.
3	(public adj space).ti,ab.
4	(park not parkin*).ti,ab.
5	greenness.ti,ab.

6	green*way.ti,ab.
7	(green adj infrastructure).ti,ab.
8	vegetation.ti,ab.
9	NDVI.ti,ab.
10	blue*space.ti,ab.
11	(water adj body).ti,ab.
12	coast*.ti,ab.
13	(blue adj infrastructure).ti,ab.
14	pond*.ti,ab.
15	water*way.ti,ab.
16	lake.ti,ab.
17	sea.ti,ab.
18	ocean.ti,ab.
19	wetland.ti,ab.
20	(matern* adj health).ti,ab.
21	pregnan*.ti,ab.
22	new*born.ti,ab.
23	"gestational age".ti,ab.
24	birth*weight.ti,ab.
25	"preterm birth".ti,ab.
26	"birth outcome*".ti,ab.
27	(infan* adj health).ti,ab.
28	"head circumference".ti,ab.
29	"gestational diabetes".ti,ab.
30	"postpartum depression".ti,ab.
31	"preeclampsia".ti,ab.
32	(pregnan* adj outcome).ti,ab.
33	(green adj space).ti,ab.
34	(blue adj space).ti,ab.
35	1 or 2 or 3 or 4 or 5 or 6 or 7 or 8 or 9 or 10 or 11 or 12 or 13 or 14 or 15 or 16 or 17 or 18 or 19 or 33 or 34
36	20 or 21 or 22 or 23 or 24 or 25 or 26 or 27 or 28 or 29 or 30 or 31 or 32
37	35 and 36

Embase search strategy

Set	Search statement
1	green*space.ti,ab.
2	(open adj space).ti,ab.
3	(public adj space).ti,ab.
4	(park not parkin*).ti,ab.
5	greenness.ti,ab.
6	green*way.ti,ab.
7	(green adj infrastructure).ti,ab.
8	vegetation.ti,ab.
9	NDVI.ti,ab.
10	blue*space.ti,ab.
11	(water adj body).ti,ab.
12	coast*.ti,ab.

13	(blue adj infrastructure).ti,ab.
14	pond*.ti,ab.
15	water*way.ti,ab.
16	lake.ti,ab.
17	sea.ti,ab.
18	ocean.ti,ab.
19	wetland.ti,ab.
20	(matern* adj health).ti,ab.
21	pregnan*.ti,ab.
22	new*born.ti,ab.
23	"gestational age".ti,ab.
24	birth*weight.ti,ab.
25	"preterm birth".ti,ab.
26	"birth outcome*".ti,ab.
27	(infan* adj health).ti,ab.
28	"head circumference".ti,ab.
29	"gestational diabetes".ti,ab.
30	"postpartum depression".ti,ab.
31	"preeclampsia".ti,ab.
32	(pregnan* adj outcome).ti,ab.
33	(green adj space).ti,ab.
34	(blue adj space).ti,ab.
35	1 or 2 or 3 or 4 or 5 or 6 or 7 or 8 or 9 or 10 or 11 or 12 or 13 or 14 or 15 or 16 or 17 or 18 or 19 or 33 or 34
36	20 or 21 or 22 or 23 or 24 or 25 or 26 or 27 or 28 or 29 or 30 or 31 or 32
37	35 and 36
