# Supplementary material for: Causal evidence of the association between green and blue spaces (GBS) and maternal and neonatal health: a systematic review and meta-analysis protocol
Source: BMJ Open. 2024 Aug 7;14(8):e082413. doi: 10.1136/bmjopen-2023-082413 (PMC11331951; doi:10.1136/bmjopen-2023-082413)
Supplement: Supplementary file 3 [file bmjopen-2023-082413.reviewer_comments.pdf]

PEER REVIEW HISTORY

BMJ Open publishes all reviews undertaken for accepted manuscripts. Reviewers are asked to complete a checklist review form (**http://bmjopen.bmj.com/site/about/resources/checklist.pdf**) and are provided with free text boxes to elaborate on their assessment. These free text comments are reproduced below.

ARTICLE DETAILS

**TITLE (PROVISIONAL)**	The causal evidence of the association between Green and Blue Spaces (GBS) and maternal and neonatal health: a systematic review and meta-analysis protocol.
**AUTHORS**	Khalaf, Rukun; Akaraci, Selin; Baldwin, Faye; Geary, Rebecca; Kolamunnage-Dona, R; Hunter, Ruth; Rodgers, Sarah

VERSION 1 – REVIEW

**REVIEWER**	Bhutta, Zulfiqar<br>SickKids Center for Global Child Health, Centre for Global Child Health
**REVIEW RETURNED**	24-Dec-2023

**GENERAL COMMENTS**	This is an important review protocol and worthy of pursuing. I am however not convinced that the authors present a compelling case for undertaking this review within a few years of the last review, especially as the intervening period had very limited studies given COVID. As is the case with Cochrane and Campbell protocols, including some information on studies identified since or those not included in past reviews would have helped.<br><br>Importantly, the definitions of Green and Blue spaces are quite high-level and lack the granularity for one to assess their importance. Will the authors review measurement methods (imaging or remote sensing versus other forms of documentation)? How will exposure and dose effects be calculated and interpreted? Mobility measures?<br><br>Given that a range of living conditions will be encountered, the protocol is silent on how the important confounding by socio-economic considerations will be taken into account?

**REVIEWER**	Liang, Jinghong<br>Sun Yat-Sen University, 6. Department of Maternal and Child Health, School of Public Health, Sun Yat-sen
**REVIEW RETURNED**	29-Feb-2024

**GENERAL COMMENTS**	The present manuscript presented with numerous methodological issues that need to be addressed. Given the significant relevance of the topic to planetary health, I am open to allowing the author an opportunity to revise the manuscript. However, if the author is unable to make substantial improvements, I may opt to reject the submission.<br>1. The statements in this study should be framed in the future tense as they outline plans for upcoming research.

	2. The primary issue with this study revolves around the challenge of establishing cause and effect relationships. It is crucial to note that while the title suggests a focus on causal relationship, the statement in manuscript lacks substantial evidence or explanation regarding causality throughout, particularly in the methodology and results sections. Moreover, the exclusion of Case control studies further complicates the ability to determine causality, as only Randomized Controlled Trials (RCTs) and high-quality cohort studies are typically capable of establishing causal relationships. Therefore, the argument for causality in this study appears to be lacking and presents a significant gap in the research.<br>3. Research in the environmental field often varies in methodology, with spatial exposure(Green and blue spaces) indicators like NDVI and NDWI commonly used in studies. Some researchers also incorporate weighted land use types to assess blue and green space exposure. This diversity in methodology can make it challenging for authors to quantify their results. Therefore, I recommend that the author explore the option of conducting a qualitative study.<br>4. The citation of references in the full text is inadequate, particularly in the methodology section where there is a lack of relevant citations to support the evidence.<br>5. Several related search terms such as Nature spaces, coasts, beaches, lakes, parks, and wetlands were not included, potentially resulting in a limited number of retrieved studies.<br>6. The author has not searched for potential studies since July 2023, and it is now March 2024. Many new relevant studies may have been produced during this time. It is recommended that the author perform a comprehensive search for relevant documents once again. Additionally, displaying the relevant search strategies for all database searches will provide a scientific and objective methodological basis.<br>7. The methods described in this article lack clarity, particularly in terms of how the included studies is integrated. The author has included both observational and experimental studies, which are distinct study design. It is important for the author to clearly outline how these different types of studies are combined. Additionally, concepts such as heterogeneity, subgroup analysis, and sensitivity analysis are not adequately introduced in the statistical methods section. Overall, the methodology section lacks objectivity and scientific rigor.<br>8. The author highlighted PRISMA as an advantage in the advantages section. Shouldn't all meta-analyses adhere to PRISMA guidelines? Can health-related issues be explored within their established themes? The author's interpretation of strengths appears to be inaccurate.<br>9. In the limitations section, the author stated that the study cannot establish a causal relationship, which seems contradictory given the emphasis on cause and effect in the theme.

VERSION 1 – AUTHOR RESPONSE

Reviewer: 1

Prof. Zulfiqar Bhutta, SickKids Center for Global Child Health

Comments to the Author:

(1.1) This is an important review protocol and worthy of pursuing. I am however not convinced that the authors present a compelling case for undertaking this review within a few years of the last review, especially as the intervening period had very limited studies given COVID. As is the case with Cochrane and Campbell protocols, including some information on studies identified since or those not included in past reviews would have helped.

Response: *Thank you for this point.*

The last relevant meta-analysis conducted on the topic was published in January 2024. However, this analysis was limited to papers published up to 2022. Since then, at least 17 studies on the topic of Green Blue Space (GBS) on maternal and/or neonatal health have been found which we aim to include in our study. Furthermore, this analysis was limited to green space measures and only some neonatal outcomes (small for gestational age, birthweight, and pre-term birth). We will include papers that investigate other outcomes such as birth head circumference and maternal outcomes (e.g. pre-eclampsia, gestational diabetes, etc).

Additionally, our study will include the effect of blue space measures. The most recent systematic review on the impact of blue space on maternal and neonatal health was published in 2020. Since then, we have identified at least 6 blue space studies to be included in our review. Example blue space exposures could include distance to a blue space and presence of blue space within a buffer region.

Thus, an updated review and meta-analysis of studies that investigate a comprehensive list of maternal and neonatal outcomes is needed. To our knowledge, our focus on the causal evidence is unique to the existing literature and a systematic review on blue space effects on maternal and/or neonatal outcomes has not been recently conducted.

We have clarified this in the Introduction and indicated some studies we aim to include in our meta-analysis and review that have not been seen elsewhere.

(see page 4, lines 84-85, lines 87-90, and page 5, lines 109-110).

(1.2) Importantly, the definitions of Green and Blue spaces are quite high-level and lack the granularity for one to assess their importance. Will the authors review measurement methods (imaging or remote sensing versus other forms of documentation)?

Response: *Thank you.*

Our study will exclude papers that employ virtual reality GBS exposure. This is because we are interested in measuring the effects of real-life GBS exposure on maternal and neonatal health. Remote sensing data and extracting images obtained from satellites are permitted as they are often used to derive metrics such as the Normalized Difference Vegetation Index (NDVI) that indicate real-world greenness exposure. This has been clarified in the text.

Furthermore, our study will also include a review of the included paper's measurement methods. We aim to do this by creating a taxonomy of the various exposure measures used.

(see page 8, lines 150-154, and pages 13, line 235-237).

(1.3) How will exposure and dose effects be calculated and interpreted? Mobility measures?

Response: *Great points, thank you.*

If possible, we will explore the linear dose-response effects for each exposure separately. We can do this by standardising the effect sizes. We anticipate separate studies could report the impact of 0.5 change in NDVI exposure or 0.1 change in NDVI reading on birthweight. Therefore, we would calculate the equivalent effect size to the smallest change (in this case 0.1 increase in NDVI) for these studies before conducting a meta-analysis. A dose response analysis will only be conducted for studies that employ similar buffer sizes (e.g. 300m). Moreover, we anticipate differing buffer types (i.e. circular or network) will make it difficult to calculate the dose response effects of distance on health outcomes.

Further clarification on the planned method of analysis has been added. Summary effect estimates are expected to be interpreted per 0.1 unit change (depending on the exposure used).

(see page 14, lines 256-258, lines 259-262).

Clarification on mobility metrics has been added. The added sentence indicates accessibility metrics such as distance between maternal residence and GBS functions as an accepted mobility measure. Moreover, we will also include measures that stipulate usage and/or visits to a GBS.

(see page 8, lines 153-154).

(1.4) Given that a range of living conditions will be encountered, the protocol is silent on how the important confounding by socio-economic considerations will be taken into account?

Response: *Thank you. This is an excellent point.*

As part of our risk of bias assessment, we include a question if confounding measures have been controlled for (Were important confounding factors measured and controlled validly and reliably?). Important co-variates include deprivation (variables could include mother's employment status, household income, or Index of Multiple Deprivation), urbanicity of mother's residence, and air pollution.

We determined these variables to be important in GBS analysis following review of the existing literature. Previous research into the impact of deprivation on health has shown that neighbourhood level deprivation can be a significant influencing factor on health. Likewise, the strength of the relationship between GBS and health is influenced by urbanicity. Those living in urban areas experience greater changes in health outcomes compared to those living in non-urban areas when changes to GBS exposure occurs. The mitigating impact of GBS on environmental pollution can result in reduced air pollution levels in areas located closer to GBS.

Studies that do not include these measures as confounders or co-variates are considered to be high risk of bias and are excluded from the meta-analysis. This has been clarified in the text.

(see page 13, line 225-227).

Stafford M, Marmot M. Neighbourhood deprivation and health: does it affect us all equally? Int J Epidemiol. 2003;32(3):357-66.

Maas J, Verheij RA, Groenewegen PP, et al. Green space, urbanity, and health: how strong is the relation? J Epidemiol Community Health. 2006;60(7):587-92.

Bixby H, Hodgson S, Fortunato L, et al. Associations between green space and health in English cities: an ecological, cross-sectional study. PLoS One. 2015;10(3):e0119495.

Reviewer: 2

Dr. Jinghong Liang , Sun Yat-Sen University

Comments to the Author:

The present manuscript presented with numerous methodological issues that need to be addressed. Given the significant relevance of the topic to planetary health, I am open to allowing the author an opportunity to revise the manuscript. However, if the author is unable to make substantial improvements, I may opt to reject the submission.

(2.1) The statements in this study should be framed in the future tense as they outline plans for upcoming research.

> ***Response:*** *Thank you. This revision has been undertaken.*

(2.2) The primary issue with this study revolves around the challenge of establishing cause and effect relationships. It is crucial to note that while the title suggests a focus on causal relationship, the statement in manuscript lacks substantial evidence or explanation regarding causality throughout, particularly in the methodology and results sections. Moreover, the exclusion of Case control studies further complicates the ability to determine causality, as only Randomized Controlled Trials (RCTs) and high-quality cohort studies are typically capable of establishing causal relationships. Therefore, the argument for causality in this study appears to be lacking and presents a significant gap in the research.

> ***Response:*** *This is a very important point. Thank you.*
>
> *To our knowledge, there are no RCTs conducted on this topic. However, in recent years, there have been more cohort studies conducted (both prospective and retrospective).*
>
> *Prospective cohort studies, experiments, and quasi experiments provide strong causal evidence because temporality can be established between the exposure and outcome. Weaker, but still valuable, causal evidence will be obtained from retrospective cohort and case control studies. This has been explained in the text.*
>
> *(see page 6, lines 125-128).*

(2.3) Research in the environmental field often varies in methodology, with spatial exposure(Green and blue spaces) indicators like NDVI and NDWI commonly used in studies. Some researchers also incorporate weighted land use types to assess blue and green space exposure. This diversity in methodology can make it challenging for authors to quantify their

results. Therefore, I recommend that the author explore the option of conducting a qualitative study.

Response: *Thank you for this comment. While a qualitative study would be beyond the scope of this review, it is well noted for exploration in future research.*

(2.4) The citation of references in the full text is inadequate, particularly in the methodology section where there is a lack of relevant citations to support the evidence.

Response: *Thank you for your comment.*

In response, we have added the below citations in our methodology section. This is reflected in the bibliography.

In the inclusion and exclusion criteria section:

These studies are cited to indicate the reason for our study design inclusion criteria.

[20] Song JW, Chung KC. Observational studies: cohort and case-control studies. Plast Reconstr Surg. 2010;126(6):2234-42.

[21] Medicine Io. 7 Scientific Evidence for Causation in the Population. Improving the Presumptive Disability Decision-Making Process for Veterans. Washington, DC: The National Academies Press; 2008 DOI: https://doi.org/10.17226/11908.

In the exposure section:

These studies were provided to reflect common exposure variables for GBS.

[23] Gascon M, Cirach M, Martínez D, et al. Normalized difference vegetation index (NDVI) as a marker of surrounding greenness in epidemiological studies: The case of Barcelona city. Urban Forestry & Urban Greening. 2016;19:88-94.

[24] Vilcins D, Sly PD, Scarth P, et al. Green space in health research: an overview of common indicators of greenness. 2022 ;doi:10.1515/reveh-2022-0083.

In the risk of bias assessment section:

These studies were included to show our reasoning for our selection of important co-variates and confounders that studies should incorporate.

[33] Stafford M, Marmot M. Neighbourhood deprivation and health: does it affect us all equally? Int J Epidemiol. 2003;32(3):357-66.

[34] Maas J, Verheij RA, Groenewegen PP, et al. Green space, urbanity, and health: how strong is the relation? J Epidemiol Community Health. 2006;60(7):587-92.

[35] Bixby H, Hodgson S, Fortunato L, et al. Associations between green space and health in English cities: an ecological, cross-sectional study. PLoS One. 2015;10(3):e0119495.

In the data synthesis section:

These references indicate guidelines for our planned analysis strategy.

[36] Popay J, Roberts, H., Sowden, A., Petticrew, M., Arai, L., Rodgers, M., Britten, N., Roen, K., and Duffy, S. Guidance on the Conduct of Narrative Synthesis in Systematic Reviews2006.

[37] Deeks JJ, Higgins, J.P.T., Altman, D.G. (editors). . Chapter 10: Analysing data and undertaking meta-analyses. In Higgins, J.PT., Thomas., J., Chandler, J., Cumpston, M., Li, T., Page, M.J., and Welch., V.A. (editors). Cochrane Handbook for Systematic Reviews of Interventions. 6.4 (updated August 2023) ed: Cochrane; 2023.

[38] Higgins JP, Thompson SG. Quantifying heterogeneity in a meta-analysis. Stat Med. 2002;21(11):1539-58.

(2.5) Several related search terms such as Nature spaces, coasts, beaches, lakes, parks, and wetlands were not included, potentially resulting in a limited number of retrieved studies.

Response: *Thank you for this point.*

We have added the advised terms to our search strategy (see pages 9-10, Table 2, and Supplementary file 2).

We opted to not include the term 'parks' because, during our search trials, this term resulted in a lot of irrelevant papers. Moreover, targeted papers in our field also often categorise these spaces as green space, or Green and Blue Space. Therefore, we think using the broader terms such as green space, etc. results in the highest specificity in our searches.

(2.6) The author has not searched for potential studies since July 2023, and it is now March 2024. Many new relevant studies may have been produced during this time. It is recommended that the author perform a comprehensive search for relevant documents once again. Additionally, displaying the relevant search strategies for all database searches will provide a scientific and objective methodological basis.

Response: *This is an excellent point.*

The study is ongoing and searches were last completed in April 2024. From our recent search, we have identified an additional 13 studies to be included in our review. We also plan to re-run our searches 1 month prior to submission for publication.

The complete search strategy for all 7 databases has been shared as a Supplementary file 2.

(2.7) The methods described in this article lack clarity, particularly in terms of how the included studies is integrated. The author has included both observational and experimental studies, which are distinct study design. It is important for the author to clearly outline how these different types of studies are combined. Additionally, concepts such as heterogeneity, subgroup analysis, and sensitivity analysis are not adequately introduced in the statistical methods section. Overall, the methodology section lacks objectivity and scientific rigor.

Response: *Thank you.*

We will combine effect estimates from both observational and experimental studies in our meta-analysis, if applicable (based on study exposure and outcomes of focus). This has been stated. (see page 14, lines 255-256).

We will define heterogeneity based on the I^2 statistics as guided by Higgins et al (2002). This has been clarified in the text (see page 14, line 267-268).

Our planned sensitivity analysis has also been expanded to include alternately excluding studies one-at-a-time in our meta-analysis to observe any changes in the pooled estimate. This has been outlined. (see page 14, line 268-269).

Our study will conduct subgroup analysis of the effect estimates based on the different exposures and buffer sizes reported. For example, the impact of changes in NDVI readings against preterm birth will be analysed and grouped based on the corresponding buffer sizes (e.g. 100m, 300m, 500m, etc.) (see page 14, line 262-264).

Higgins JP, Thompson SG. Quantifying heterogeneity in a meta-analysis. Stat Med. 2002;21(11):1539-58.

(2.8) The author highlighted PRISMA as an advantage in the advantages section. Shouldn't all meta-analyses adhere to PRISMA guidelines? Can health-related issues be explored within their established themes? The author's interpretation of strengths appears to be inaccurate.

Response: *Thank you, your point is noted.*

This has been removed as a strength. Instead, a strength of our review will be the inclusion of papers published since 2022 (up to 17 papers). This has been clarified.

(see page 15, line 289).

(2.9) In the limitations section, the author stated that the study cannot establish a causal relationship, which seems contradictory given the emphasis on cause and effect in the theme.

Response: *Thank you.*

For clarification, we expect difficulty in establishing a causal relationship between blue space and maternal/neonatal health outcomes. This is because we expect there will be substantially less studies that include blue space as an exposure of interest compared to green space studies. Thus, we anticipate we will be unable to carry out a meta-analysis with blue space studies. Instead, we will carry out narrative synthesis of blue space studies.

Based on the anticipated number of green space studies, we anticipate being able to establish a causal relationship between green space and neonatal health.

(see page 3, lines 64-65, and page 15, lines 293-295).

Reviewer: 1

Competing interests of Reviewer: No competing interests

Reviewer: 2

Competing interests of Reviewer: No.

VERSION 2 – REVIEW

**REVIEWER**	Bhutta, Zulfiqar<br>SickKids Center for Global Child Health, Centre for Global Child Health
**REVIEW RETURNED**	28-Jun-2024

**GENERAL COMMENTS**	Thanks. I think the responses and edits are reasonable. I have no further questions or concerns

VERSION 2 – AUTHOR RESPONSE

Reviewer: 1

Prof. Zulfiqar Bhutta, SickKids Center for Global Child Health

Comments to the Author:

Thanks. I think the responses and edits are reasonable. I have no further questions or concerns.

Reviewer: 1

Competing interests of Reviewer: No competing interests.
